# A splice donor in *E6* influences keratinocyte immortalization by beta-HPV49

**DOI:** 10.1128/jvi.01640-24

**Published:** 2025-01-22

**Authors:** Tina M. Rehm, Thomas Iftner, Frank Stubenrauch

**Affiliations:** 1 Institute for Medical Virology and Epidemiology of Viral Diseases, University of Tuebingen, Tuebingen, Germany; College of Agriculture & Life Sciences, University of Arizona, Tucson, Arizona, USA

**Keywords:** beta-HPV, E6, RNA splicing, viral transcriptome

## Abstract

Human papillomaviruses (HPV) from the genus beta have been implicated in the development of cutaneous squamous cell cancer in *epidermodysplasia verruciformis* and organ transplant patients. In contrast to alpha-high-risk HPV, which cause ano-genital and oropharyngeal cancers, beta-HPV replication is not well understood. The beta-HPV49 transcriptome was analyzed by RNA sequencing using stable keratinocyte cell lines maintaining high levels of extrachromosomally replicating E8- genomes, which can be established due to a lack of the viral E8^E2 repressor protein. This analysis indicated the presence of four transcription start sites, two polyadenylation signals, and splice donor (SD) and acceptor sites consistent with the conserved gene expression patterns of animal and human PV. Surprisingly, a novel SD in the *E6* oncogene (SD217) was identified resembling the SD in *E6* of carcinogenic alpha-HPV. Mutation of SD217 enhanced E6 protein expression but had no influence on the growth of keratinocytes transduced with retroviral HPV49 E6 and E7 expression vectors. Inactivation of SD217 in the context of the HPV49 wild-type genome did not enable immortalization and prevented immortalization in the context of the E8- genome. The analysis of SD217 mutant genomes revealed a strong down-regulation of SD217 usage, but only weak effects on other viral transcripts. This suggests that SD217 does not contribute to immortalization by modulating viral gene expression. Usage of SD217 is increased in immortalized E8- cell lines compared with transiently transfected cells, which may indicate that long-term extrachromosomal maintenance requires reduced E6 protein levels.

**IMPORTANCE:**

High-risk (hr) human papillomaviruses (HPV) from the genus alpha cause ano-genital and oropharyngeal cancers, whereas beta-HPV have been implicated to cause skin cancer in *epidermodysplasia verruciformis* and organ transplant patients. In contrast to alpha hr-HPV, the replication cycle of beta-HPV is not very well understood. Transcriptional profiling of beta-HPV49 by RNA sequencing reveals transcription start sites and splice sites conserved among HPV. Surprisingly, a splice donor site in the *E6* oncogene (SD217), previously only described for hr-HPV, was identified that controls E6 oncoprotein levels and is required for immortalization of keratinocytes by the HPV49 genome.

## INTRODUCTION

Human papillomaviruses (HPV) are a large virus family, which are grouped into five genera: alpha-, beta-, gamma-, mu-, and nu-PV. Persistent infections with high-risk (hr)-HPV (HPV16, 18, 31, 33, 35, 39, 45, 51, 52, 58, and 59) from the genus alpha result in annually 630,000 new cancer cases at different sites like the cervix uteri, anus, larynx, oropharynx, oral cavity, penis, vulva, and vagina (1). On the other hand, HPV from the genus beta have been implicated in the development of cutaneous squamous cell cancer in *epidermodysplasia verruciformis* and organ transplant patients (2–4).

The organization of the covalently closed HPV dsDNA genome of ~8 kbp is highly conserved. It is divided into three parts: the non-coding upstream regulatory region (URR), which encompasses transcription control elements, transcription start sites (TSS) and the origin of replication, the early region, and the late region. The early region encodes the E1, E2, E4, E6, and E7, and in the case of alpha-HPV, E5, proteins. E1 and E2 are DNA-binding proteins required for genome replication (5, 6). E2 has in addition transcription-modulating and genome partitioning activities (6). E6 and E7 modulate cell death, differentiation, proliferation, and DNA repair pathways and also have immune-evasive activities (7). Furthermore, RNA splicing results in the generation of the conserved viral fusion proteins E1^E4 and E8^E2. E1^E4 consists of a short peptide from E1, which provides a start codon and is fused to the E4 gene. Despite being encoded in the early region, E1^E4 is a late, non-structural protein involved in the productive replication stage (8). In E8^E2, the *E8* gene is spliced to the second half of *E2*, which results in a repressor protein limiting viral gene expression and genome replication (9). The late region encodes the L1 and L2 capsid proteins. Polyadenylation signals flank the early and late regions in order to allow adding polyA-tails to all viral transcripts. Interestingly, the vast majority of HPV transcripts are polycistronic and are derived from different TSS located in the URR and the beginning of the early region and thus differ in their 5′-extension (10). A highly conserved feature of all HPV is a promoter at the very end of the URR in front of *E6* which drives the expression of a precursor transcript encompassing the complete early region. Remarkably, only hr-HPV and phylogenetically very closely related HPV use facultative splice donor (SD) and splice acceptor (SA) sites in *E6* to generate truncated E6 proteins, labelled E6*, from this precursor transcript (10). Whereas the function of E6* proteins remains unclear, removal of the facultative E6 intron enables efficient translation of the downstream E7 gene (10). Furthermore, an E6*, *E7*, *E1*, *E2*, and *E5* transcript is thought to be the major source for the E1 replication protein at early stages of the hr-HPV infection (11–13).

A key feature of hr-HPV E6 and E7 proteins is the efficient immortalization of normal human keratinocytes (NHK), the target cell for HPV *in vivo*. Only few non-hr-HPV such as beta-HPV38, 49, 75, and 76 E6 and E7 share the ability to immortalize NHK when expressed from retroviral vectors (14–17). Surprisingly, complete HPV38 or HPV49 genomes are unable to immortalize NHK despite detectable *E6* and *E7* expression (18, 19). However, the HPV49, but not the HPV38 genome, becomes immortalization competent when the viral E8^E2 repressor protein (E8-) is genetically inactivated (18, 19). This is due to greatly increased E6 and E7 transcript levels caused by enhanced genome replication (19).

In contrast to hr-HPV, the structures of beta-HPV transcripts are underexplored. Previous studies have focused on HPV5 and 8 using biopsy material or non-keratinocyte cell culture models (20–23). This has revealed commonalities and differences to hr-HPV: similar to hr-HPV (and other PV), TSS are found in front of *E6*, within *E7*, and within *E1* in front of *E8*. Furthermore, conserved SD sites at the beginning of *E1* and in the *E8* region and SA sites upstream of *E2*, in the *E2/E4* region, and immediately upstream of L1 can be found (20–23). In contrast to hr-HPV, but consistent with other HPV, a TSS in *E6* has been described for HPV5 and 8 (21, 22). Moreover, a TSS in the URR and a SD site in the URR are used that are absent from hr-HPV and other alpha-HPV but also present in HPV1 (20, 23, 24). Based on this information, we previously predicted conserved splice sites in HPV38 and 49 and confirmed them by quantitative PCR (qPCR) analysis and sequencing in transfected NHK and stable HPV49 E8- keratinocyte cell lines (18, 19).

We now describe a viral transcriptome analysis by RNA-sequencing of stable HPV49 E8- cell lines, which maintain almost exclusively autonomously replicating genomes. Our data confirm previous studies but also reveal novel SD and SA sites. Remarkably, HPV49 uses a SD site in *E6*, which controls E6 protein levels and is important for immortalization by the HPV49 E8- genome.

## RESULTS

### Transcriptome analysis of HPV49 E8- cell lines reveals known and novel splice junctions

**Fig 1:**
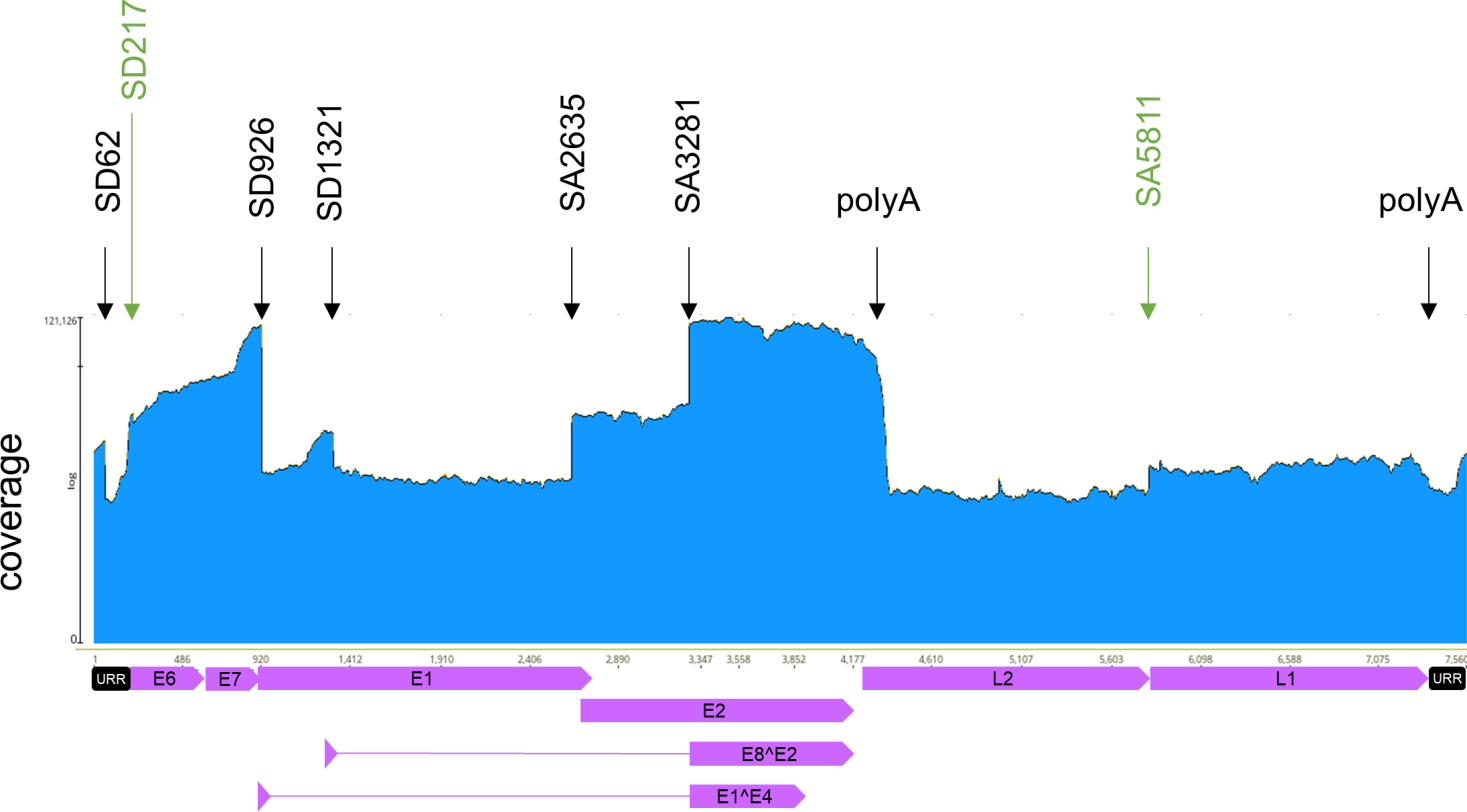
Visualization of read coverage of RNA sequencing data mapped to the HPV49 reference genome linearized at nt. 1. HPV49 open reading frames (ORFs), the URR, and spliced genes are indicated below. Splice signals (SD; SA) followed by the nucleotide number are indicated by arrows and are shown in black for previously identified sites and in green for major novel sites. The positions of consensus poly adenylation (polyA) sites are indicated by arrows. Data were obtained from three different cell lines derived from different donors. A log10 scale is used on the y-axis.

**Fig 2:**
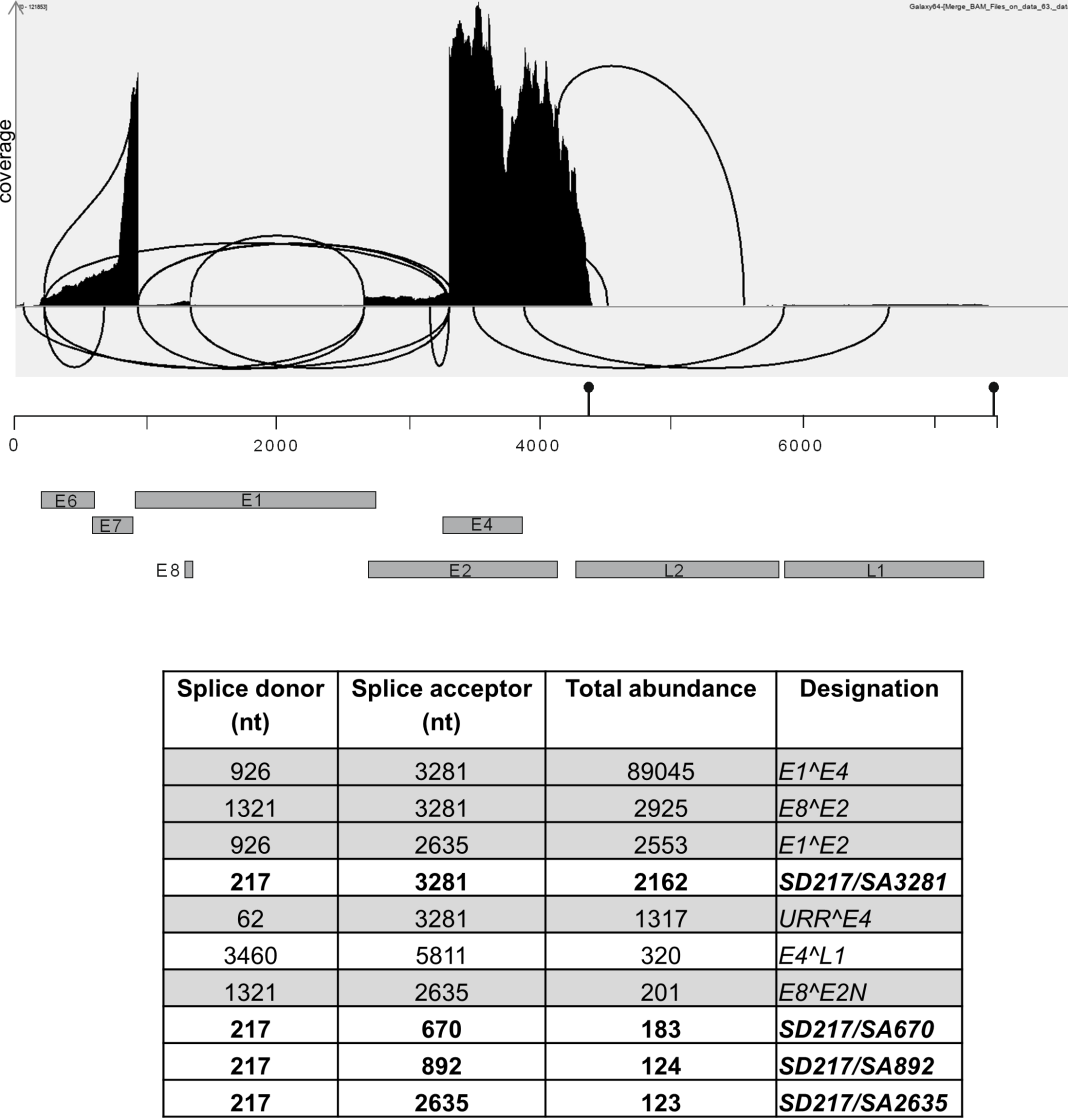
Exon junctions from RNA sequencing data of three different HPV49 E8- genome containing cell lines were identified (HISAT2 aligner, enable spliced alignment), displayed and counted with the integrated genome viewer, and are depicted as a Sashimi plot. Read abundances on the y-axis are shown on a linear scale. Only splice junctions with >100 supporting reads are shown. Only splice junctions with more than 100 reads and present in all three cell lines are listed in the table. The linearized HPV49 reference genome with ORFs is shown below. The positions of consensus poly adenylation sites are indicated by pins.

Total RNA from three previously characterized HPV49 E8- cell lines maintained in monolayer culture was isolated, poly-A-selected, and then subjected to short-read RNA sequencing. Mapping of the viral reads to the HPV49 reference genome revealed that 971,666 viral reads representing 0.6% of total reads were detected (Fig. 1 and 2). The sharp signal decreases in the URR, at the beginning of *E1*, at the end of *E8*, in *E1* before the beginning of *E2*, and in the *E2/E4* region map to the previously mapped SA and SD sites SD62, SD926, SD1321, SA2635, and SA3281. The highest coverage was observed for the 3′-end of *E7* to SD926 in *E1* and from SA3281 in the *E2/E4* region to the beginning of *L2* (Fig. 1 and 2). Medium coverage was observed from the beginning of *E6* to the 5′-end of E7 and from SA2635 to SA3281 (Fig. 1 and 2). Low coverage was observed in the URR from nt. 7,508 to SD62, from nt. 1,177 upstream of *E8* to SD1321, and in the *L1* gene (Fig. 1 and 2). Very low coverage was observed in *E1* with the exception of the region covering *E8*, *L2*, and parts of the URR between the end of *L1* and downstream of SD62 to the start of *E6* (Fig. 1 and 2). Taken together, these results confirm previously mapped splice sites and also suggest that four TSS are used: in the 5′-URR, in front of *E6*, in the 3′-part of *E7*, and in front of *E8*, which is consistent with previous findings for other HPV. Furthermore, the sharp signal decrease at the beginning of *L2* and downstream of *L1* is consistent with the presence of polyadenylation signals at nt. 4,323–4,328 (aataaa) and nt. 7,365–7,370 (aataaa). The analysis of splice junction reads revealed both known and new SD and SA sites (Fig. 2): SD62 to SA2635 (*URR^E2*), SD62 to SA3281 (*URR^E4*), SD926 to SA2635 (*E1^E2*), SD926 to SA3281 (*E1^E4*), SD1321 to SA2635 (*E8^E2N*), and SD1321 to SA3281 (*E8^E2*), and their abundances are consistent with published qPCR results (Fig. 2; [19]). A novel splice junction linked SD3460 in the *E2/E4* region to SA5811 at the beginning of *L1* (*E4^L1*), resembling conserved *L1*-encoding transcripts in other PV. Interestingly, a novel SD site in *E6* (SD217) was linked to the known SA3281 (SD217/SA3281), or SA2635, but also to novel SA670 and SA892 sites in *E7* (Fig. 2). Since SD sites in *E6* have only been reported for hr-HPV, but not other HPV, and the number of reads for the SD217/SA3281 splice junction were only slightly less abundant than *E8^E2* or *E1^E2*, it was possible that SD217 contributes to viral replication or immortalization and was therefore further analyzed.

### SD217 is used by HPV49 wt and E8- genomes

**Fig 3:**
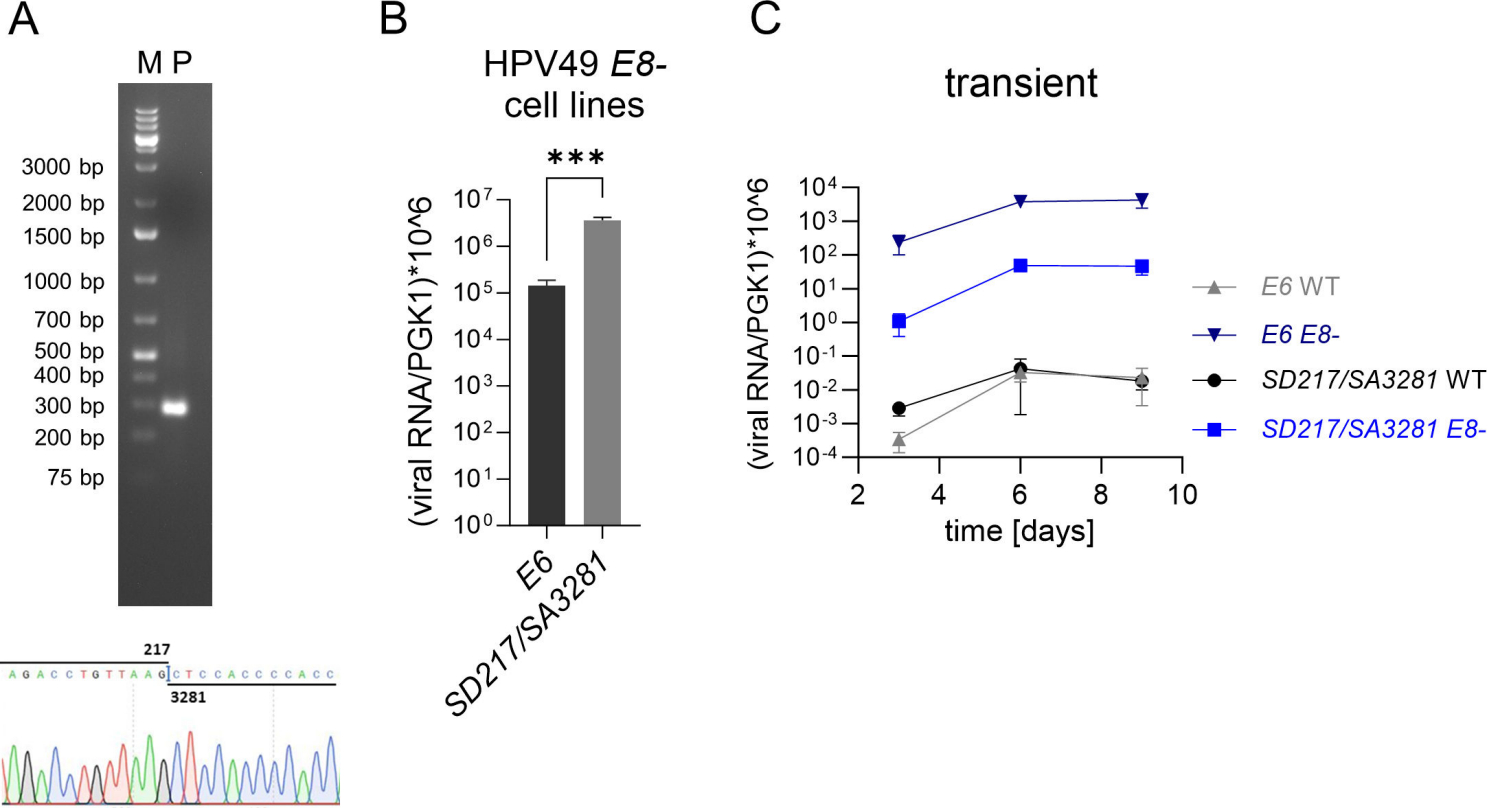
(**A**) Agarose gel analysis of the RT-qPCR product (**P**) to detect the SD217/SA3281 splice junction in total RNA isolated from HPV49 E8- cells. A size marker (**M**) is shown on the left. A partial sequence of the RT-qPCR product obtained is shown below. qPCR analysis of unspliced *E6* and spliced SD217/SA3281 transcripts in stable HPV49 E8- cell lines using total RNA (**B**) or in NHK transiently transfected with HPV49 wt or E8- genomes harvested 3, 6, or 9 days post transfection using polyA^+^-enriched RNA (**C**). (**B**) *n* = 9, paired t-test. (***P < 0.001); (**C**) *n* = 4. Error bars indicate the SEM.

We first confirmed the use of the most abundant SD217/SA3281 junction in HPV49 E8- cell lines. A reverse transcription (RT)-qPCR analysis using primers upstream of *E6* and downstream of SA3281 in *E4* and total RNA from HPV49 E8- cell lines revealed one major amplification product of 291 bp and direct sequencing analysis confirmed the use of the SD217/SA3281 junction (Fig. 3A). The levels of spliced SD217/SA3281 transcripts are significantly higher than those of unspliced *E6* transcripts (Fig. 3B). To exclude that SD217 is only used in the absence of E8^E2, HPV49 wild-type (wt) and E8- genomes were transiently transfected into NHK and the amounts of spliced SD217/SA3281 and unspliced *E6* transcripts were determined (Fig. 3C). Both wt and E8- genomes expressed SD217/SA3281 transcripts albeit to different amounts (Fig. 3C). Whereas the levels of SD217/SA3281and *E6* transcripts were similar in wt-transfected cells, much higher levels of unspliced *E6* than spliced SD217/SA3281transcripts were present in E8-transfected cells. This confirms that splicing at SD217 is not restricted to E8- genomes. It also indicates that the loss of E8^E2 not only increases the amounts of spliced and unspliced *E6* transcripts but also influences their ratio. Furthermore, the comparison of being transiently transfected with being stably maintained E8- genomes reveals that the ratio of unspliced to spliced *E6* changes during the immortalization process, suggesting that splicing at SD217 is a regulated event.

### SD217 inhibits E6 protein expression

**Fig 4:**
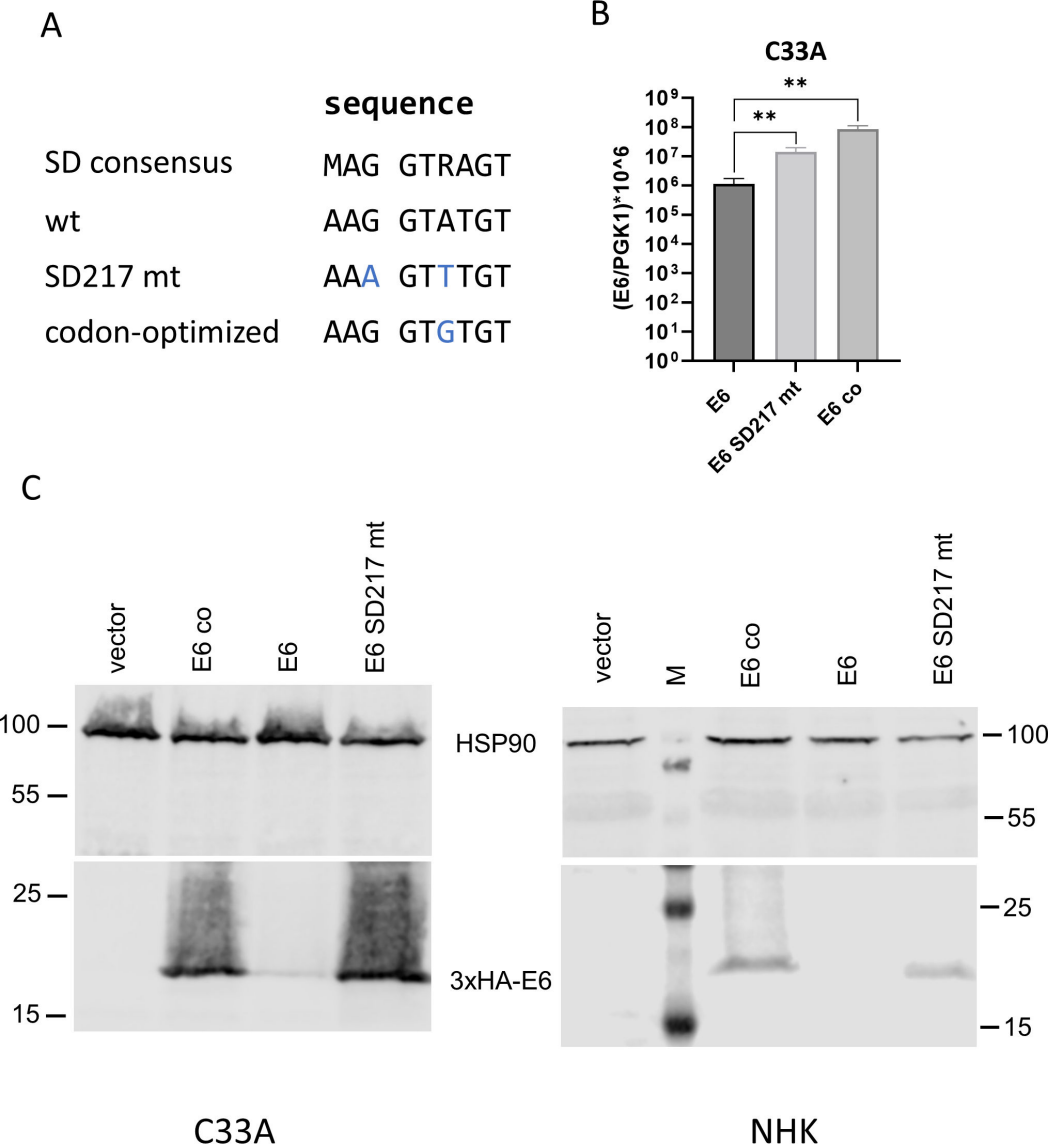
(**A**) Sequence alignment of the SD consensus (25), HPV49 SD217 wt, SD217 mt, and codon-optimized (co) E6 sequences. (**B**) qPCR analysis of unspliced *E6* transcripts using polyA^+^-enriched RNA isolated from C33A cells transfected with pSG HPV49 3xHA E6 (**E6**), pSG HPV49 3xHA E6 SD217 mt (E6 SD217 mt), or pSG HPV49 3xHA E6co (E6 co) plasmids. *n* = 5, statistical significance was determined by a ratio-paired t-test (***P < 0.01). (**C**) Immunoblot analysis of C33A (left panel) or NHK (right panel) transfected with the empty vector or expression vectors for 3xHA-tagged wt E6, E6 co, or E6 SD217 mt is shown below. E6 was detected with an anti-HA antibody, and HSP90 was used as a loading control. In the right panel, a pre-stained protein marker (**M**) is shown.

Splicing in *E6* could regulate the amounts of full-length E6 protein and thereby influence E6-dependent activities such as the immortalization of NHK. We first tested if SD217 regulates the expression of full-length E6 protein. SD217 matches the SD consensus sequence, and we therefore mutated G217 and A220 to disrupt SD217 without changing the E6 coding sequence (25) (Fig. 4A). The E6 sequence was cloned into an expression plasmid and tagged with a triple HA epitope at the N-terminus to enable protein detection. In addition to wt and SD217 mutant (mt) *E6*, a codon-optimized (co) *E6* that only minimally changes the SD217 sequence was included (Fig. 4A). We first determined the amount of *E6* transcripts by qPCR in polyA^+^-enriched RNA isolated from transfected C33A cells (Fig. 4B). This revealed significantly increased E6 transcripts in pSG HPV49 3xHA-E6 SD217 mt- and pSG HPV49 3xHA E6 co compared with pSG HPV49 3xHA E6-transfected cells indicating that mutation of SD217 or codon optimization increase *E6* expression. Immunoblot analyses of transfected C33A or NHK cells revealed that very little E6 protein was expressed from the wt sequence (Fig. 4C). In contrast, E6 protein from the SD217 mt vector was abundantly expressed. Similarly, E6 protein from E6 co was expressed at levels comparable to SD217 mt. These data strongly suggest that SD217 limits the expression of *E6* transcripts and consequently the amounts of E6 protein both in immortalized and normal keratinocytes. Furthermore, the high-level expression of E6 protein from E6 co indicates that either the minor change of the SD consensus sequence or, more likely, the change to more frequent codons and cryptic regulatory elements contributes to E6 protein expression.

### Mutation of SD217 does not influence growth of keratinocytes retrovirally-transduced with HPV49 E6 and E7

**Fig 5:**
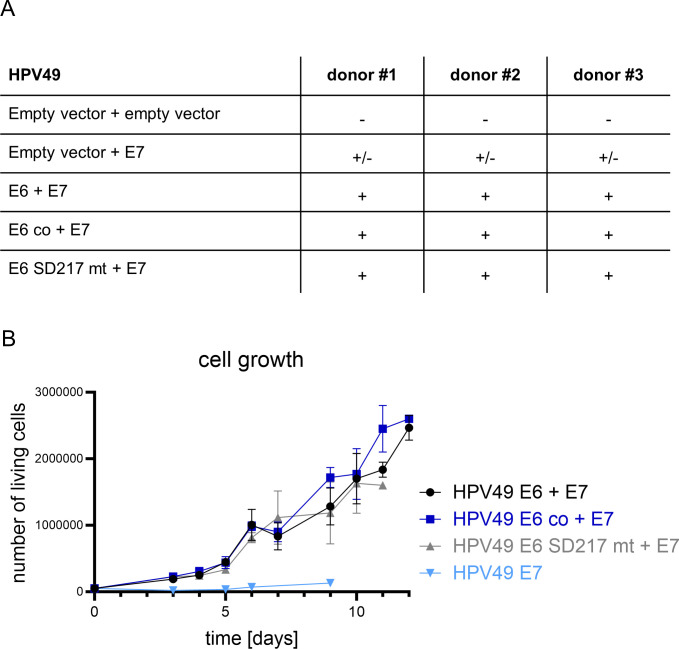
(**A**) Overview of the immortalization capabilities of HPV49 E6 and E7 in NHK. NHK from three different donors were transduced with combinations of recombinant retroviruses as indicated in the table. + indicates immortalization, +/- indicates prolonged life span, and - indicates no prolonged life span. (**B**) Growth curves of cell lines immortalized with HPV49 E7 and with or without different E6 (wt, co, and SD217 mt). Data are derived from five to nine independent experiments for E6/E7 cell lines. The growth curve of HPV49 E7 is derived from one experiment. Error bars indicate the SEM.

Expression of the HPV49 *E6* and *E7* genes has been previously shown to immortalize NHK (15). To evaluate, if splicing in *E6* modulates immortalization or the growth properties of transduced NHK, the different *E6* genes were cloned into pLXSN-neo and HPV49 *E7* in pMSCV-puro retroviral vectors. NHK from three different donors were infected with the different combinations of recombinant retroviruses as indicated in Fig. 5A and drug selected. As expected, the combination of wt E6 and E7 but also with E6 SD217 mt or E6 co resulted in immortalized cell lines without notable differences. In contrast, E7 alone extended only the life span but did not give rise to immortalized cell lines. To our knowledge, this reveals for the first time that HPV49 E7 alone does not immortalize NHK. RNA sequencing of wt E6 and E7 expressing keratinocytes indicated that SD217 is used and linked to different cryptic splice sites in the pLXSN vector suggesting that SD217 is also used in the absence of HPV49 SA sites (Fig. S1). To analyze the different cell lines in more detail, cell growth was monitored over several days (Fig. 5B). Consistent with the findings above, cells expressing only E7 barely grew in the observation period and the experiment could only be performed once. On the other hand, no growth differences were obvious between the different E6 vectors in combination with E7. This suggests that the growth of NHK is not influenced by different E6 protein levels.

### Mutation of SD217 prevents immortalization by HPV49 E8- genomes

We reported previously that the HPV49 wt genome was unable to immortalize NHK, whereas the E8- genome was capable in an E6- and E7-dependent manner (19). Since the inactivation of E8^E2 greatly increases *E6* and *E7* transcription (19) (Fig. 3B and C), it was possible that the inactivation of SD217 would increase E6 protein amounts and render the HPV49 genome immortalization competent in the presence of E8^E2. We therefore introduced the SD217 mt into the wt genome (SD217mt) or into the E8- background (E8-/SD217mt) and carried out NHK immortalization assays using cells from two different donors (Table 1). Interestingly, SD217mt, but also E8-/SD217mt, in contrast to E8- genomes did not immortalize NHK. This suggests that the putative increase in E6 protein levels is not sufficient to overcome the restrictions by E8^E2. Furthermore, the loss of immortalization in the context of the E8- genomes indicates either that a further increase in E6 levels is incompatible with immortalization or that SD217 provides additional functions important for immortalization.

**TABLE 1:** NHK immortalization assays with different HPV49 genomes

HPV49 genome	Donor #1	Donor #2
wt	−	−
SD217 mt	−	−
E8-	+	+
E8-/SD217 mt	−	−

^*a*^ Outgrowth of immortalized cell lines or lack thereof is symbolized by + or −, respectively.

**Fig 6:**
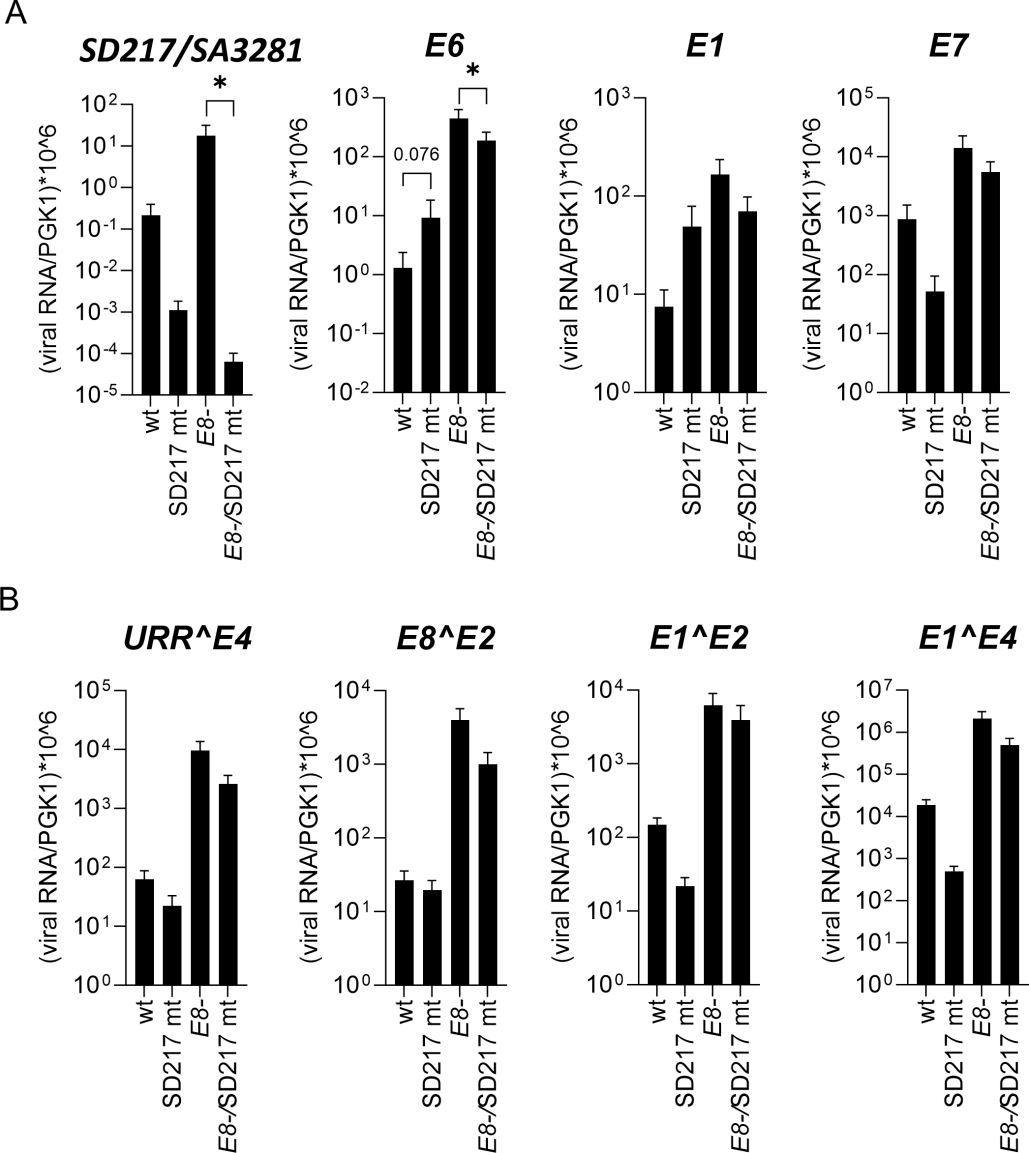
Viral gene expression analysis of NHK transiently transfected with different HPV49 genomes as indicated by qPCR using *PGK1* as a reference gene. (**A**) PolyA^+^-enriched RNA was isolated 6 days post transfection and analyzed for *SD217/SA3281*, *E6*, *E1*, and *E7* transcripts. (**B**) Total RNA was isolated 6 days post transfection and analyzed for *URR^E4*, *E8^E2*, *E1^E2*, and *E1^E4* transcripts. Values were calculated from plasmid standard curves. Data are derived from five (**A**) or nine (**B**) independent transfection experiments. Statistical significance was determined using a ratio-paired t-test using wt/ E8- as reference for the respective SD217 mt (*P < 0.05). Error bars indicate the SEM.

The corresponding SD in hr-HPV *E6* controls not only the amounts of E6 and E7 proteins but also the amount of E1 protein and thereby genome replication (11–13). Since E1 is also required for immortalization by E8- genomes (19), an interference with E1 expression remained a plausible explanation for the lack of immortalization by E8-/SD217mt genomes. Our previous study has shown that the inactivation of E1 reduces viral gene expression (19). We therefore transiently transfected NHK with wt, SD217mt, E8-, or E8-/SD217mt genomes and determined viral gene expression by qPCR 6 days post transfection. To facilitate the analysis of unspliced *E6*, *E7*, *E1*, and spliced SD217/SA3281 transcript levels after genome transfection, polyA^+^-enriched RNA was used (Fig. 6A). The E8-/SD217 mt genome expressed significantly lower SD217/SA3281 transcript levels than E8- genomes consistent with the inactivation of SD217. SD217/SA3281 levels were also lower in SD217 mt-transfected cells compared with the wt, but this did not reach statistical significance, most likely due to the overall lower transcript levels from wt and SD217 mt genomes. E8-/SD217 mt genomes expressed significantly lower *E6* levels (2.4-fold) than E8- genomes, whereas SD217 mt genomes showed a trend toward increased *E6* expression in comparison to the wt as expected upon inactivation of SD217 (Fig. 6A). Unspliced *E1* transcripts showed trends similar to *E6* and were reduced by E8-/SD217 mt and increased by SD217 mt compared with E8- and wt, respectively, but this did not reach statistical significance. In contrast, unspliced *E7* and spliced *URR^E4*, *E8^E2*, *E1^E2*, and *E1^E4* transcripts showed trends for lower expression from SD217 mt and E8-/SD217 mt genomes compared with wt and E8- genomes but this did not reach statistical significance (Fig. 6A and B). Taken together, these data confirm that the inactivation of SD217 greatly reduces SD217/SA3281 levels from E8- genomes consistent with its functional inactivation. However, with the exception of unspliced *E6*, which is slightly reduced, all other transcripts are unchanged with a trend toward a slight reduction. These surprisingly mild transcriptional phenotypes do not provide strong evidence that SD217 contributes to viral genome replication.

## DISCUSSION

**Fig 7:**
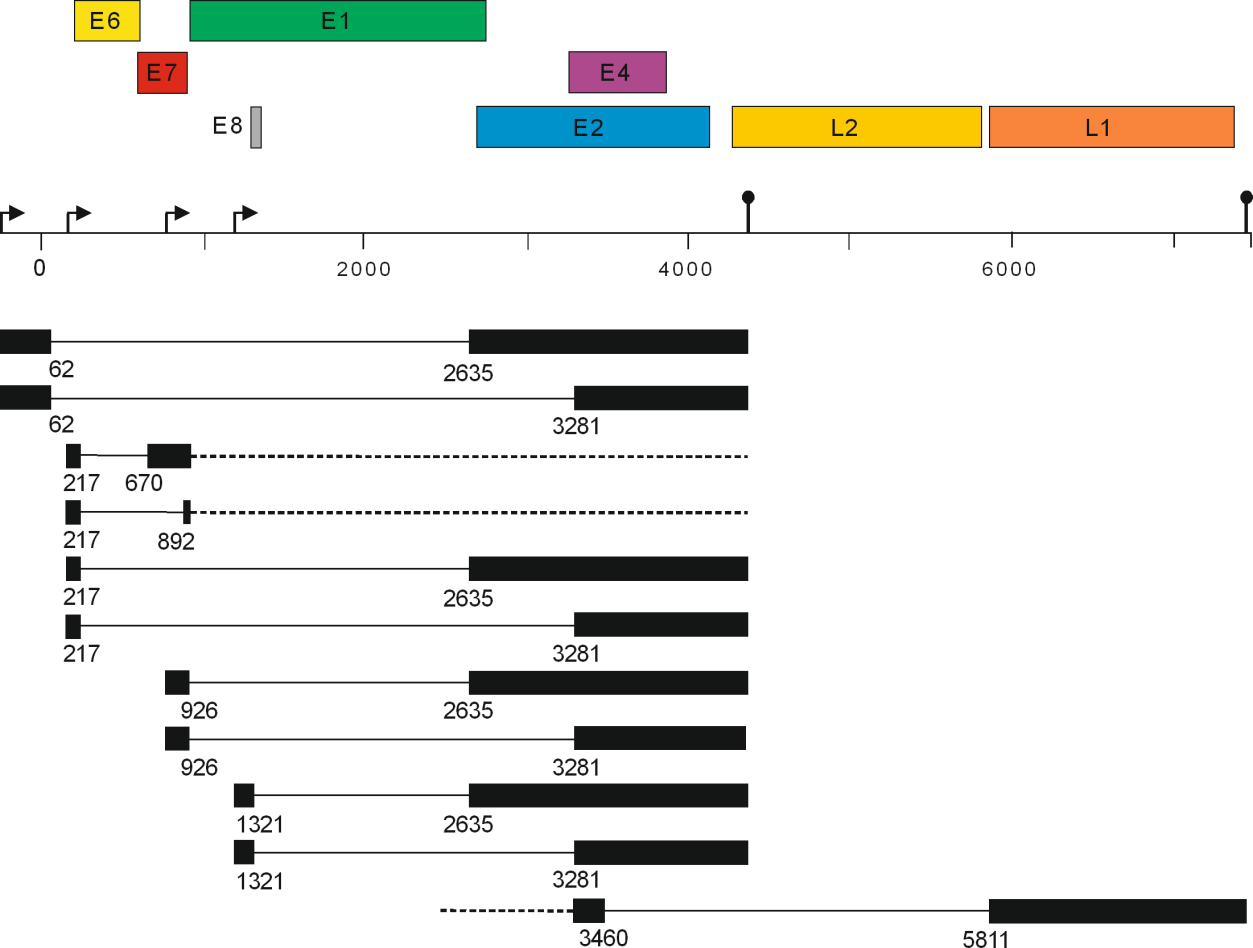
Transcript map of HPV49. The HPV49 genome was linearized at nt. 7,500 to enable the depiction of transcripts initiated at the putative promoter in the 5′URR. ORFs are shown above the linearized genome. Potential TSS are depicted by arrows and polyadenylation signals by pins. Spliced transcripts are shown below the linearized genome. Solid lines represent introns removed from spliced transcripts and dotted lines indicate unknown 5′- or 3′-extensions. Numbers indicate the last or first nt. of the exon.

Transcriptome analysis of the HPV49 E8- genome in stable keratinocyte cell lines by RNA sequencing suggested TSS in the 5′ of the *URR*, in front of *E6*, in the 3′-region of *E7*, and in front of *E8*, polyadenylation signals at the end of the early and late regions as well as splice sites in the URR (SD62), at the beginning of *E1* (SD926), at the end of *E8* (SD1321), in front of *E2* (SA 2635), and in the *E2/E4* region (SA3281) consistent with the conserved expression patterns of animal and human PV (Fig. 7).

Interestingly, the highest coverage was obtained for the 3′-end of *E7* until SD926 and from SA3281 to the end of the early region. In combination with qPCR analyses, this strongly suggests that the most abundant HPV49 transcript is a mono-cistronic *E1^E4* encoding RNA initiated at TSS in *E7* downstream of nt. 786 and ending at the early polyadenylation signal. This transcript strongly resembles the abundant *E1^E4*, *E5* transcript expressed by hr-HPV from the differentiation-dependent, major late promoter in *E7* in differentiated keratinocytes (26, 27). qPCR analyses reveal that the levels of the *E1^E4* splice junction are also far more abundant than signals for unspliced *E7* not only in E8- but also in wt-transfected cells (Fig. 6A and B), suggesting that the promoter in *E7* is also active on wt genomes in cells maintained as submerged monolayer cultures. Consistent with this, *E1^E4* transcript levels are also higher than *E7* transcript levels in human keratinocytes transfected with HPV8 and 38 genomes (18). Furthermore, the corresponding HPV5 promoter is also the most active one in U2OS cells maintained in monolayer culture (22). Taken together, this strongly suggests that the promoter in beta-HPV *E7* is more active than the *E6* promoter in undifferentiated cells, which is different from hr-HPV and might indicate fundamental differences in the viral replication cycle between hr-HPV and beta-HPV.

Surprisingly, SD217, a SD site in *E6*, was identified, which has been up to now only reported for hr-HPV and phylogenetically closely related types but not for other HPV. SD217 is mainly linked to SA3281 (SD217/SA3281), the preferred SA in the HPV49 genome, but also to SA670 and SA892 in *E7* (Fig. 2 and 7). In contrast to hr-HPV, where the SD in *E6* is mainly linked to SA in *E6* and thus creating truncated E6* proteins, the SD217/SA3281 splice junction would generate a fusion of E6 with the third ORF in the *E2/E4* region resulting in a fusion protein of E6 aa1-6 and 48 residues from the third ORF. Comparable splice junctions in hr-HPV16 and 18 could produce an E6 fusion with six and two residues, respectively, from the third ORF, whereas HPV31 would produce an E6^E4 fusion protein. This indicates that an E6 fusion protein with an extended third ORF from the *E2/E4* region is not highly conserved among HPV. Furthermore, the expression of HPV49 E6 and E7 from different vectors in the absence of other viral sequences results in the efficient immortalization of human keratinocytes (Fig. 5) making it unlikely that the putative E6 fusion protein expressed from SD217/SA3281 is a major contributor to the immortalization process. Interestingly, inactivation of SD217 enhanced E6 protein levels from expression vectors suggesting that one function is limiting E6 protein amounts comparably to hr-HPV. However, keratinocytes immortalized by retroviral transduction of HPV49 E6 and E7 did not reveal differential growth properties when SD217 was inactivated, suggesting that increased E6 protein levels do not influence cell growth. However, we cannot exclude that cells with high E6 protein levels do not survive the drug selection process.

Surprisingly, inactivation of SD217 in the E8- genome interfered with its immortalization capability and did not allow immortalization of wt genomes. The latter phenotype is most likely due to the fact that even upon inactivation of SD217 the amounts of unspliced *E6* transcripts are much lower than those produced by E8- genomes (Fig. 3C). Splicing in hr-HPV *E6* not only regulates E6 and E7 protein levels but also is required for E1 protein production and genome replication (11–13, 28). Interestingly, the SD217 mt in the context of the E8- genome, despite completely inhibiting the expression of SD217/SA3281 transcripts, neither increased unspliced *E6* nor *E1* transcript levels in transient transfection assays (Fig. 6). However, the levels of unspliced *E6* and *E1* transcripts greatly exceed SD217/SA3281 levels (Fig. 6), and thus, a further increase might be too small to be detected. Nevertheless, these findings do not support the idea that SD217 is a major regulator of E1 levels. All other evaluated transcripts, which can be derived from promoters in the URR, *E7*, or *E1*, also do not change significantly suggesting that the inactivation of SD217 has no direct impact on individual HPV49 promoters or genome replication. However, the trend to generally decreased transcript levels may indicate a subtle effect on replication.

Notably, the wt displays approximately an equal ratio between unspliced *E6* and SD217/SA3281 transcripts, whereas E8- genomes show dramatically more unspliced *E6* than SD217/SA3281 in short-term assays suggesting that the loss of E8^E2 influences splicing of viral transcripts (Fig. 3). Interestingly, in immortalized keratinocytes maintaining replicating E8- genomes, the spliced SD217/SA3281 transcript is far more abundant than unspliced *E6*. This suggests that usage of SD217 is either regulated differently at early time points and in the maintenance phase or that there is selective pressure against high levels of E6 during immortalization.

## MATERIALS AND METHODS

### RNA sequencing

Total RNA was extracted from three different HPV49 E8- positive cell lines with the RNAeasy kit from Qiagen, including an on-column DNAse digestion step (RNAse-free DNAse, Qiagen). Library preparation and RNA sequencing were done by the NGS Competence Center Tübingen, Institute for Medical Genetics and Applied Genomics. The RNA concentration was measured with the Qubit Fluorometric Quantitation and the RNA Broad-Range Assay (Thermo Fisher Scientific). The RNA integrity number (RIN) was analyzed with the Fragment Analyser 5300 and the fragment analyzer RNA kit (Agilent Technologies) and was sufficient (RIN > 8). The mRNA was enriched from 200 ng total RNA with the polyA-purification kit (NEBNext Poly(A) mRNA Magnetic Isolation Module, NEB). The library was prepared using the NEBNext Ultra II Directional RNA Library-Prep Kit for Illumina (NEB) according to the manufacturer’s instructions. To determine the molarity, the size of the library (about 400 ng) was analyzed with the Fragment Analyzer 5300 and the Fragment Analyzer DNA HS NGS Fragment Kit (Agilent Technologies). The concentration was determined (>5 ng/µL) with the Qubit Fluorometric Quantitation and the dsDNA High sensitivity assay (Thermo Fisher Scientific).

The library was denatured according to the manufacturer’s instructions, diluted to 270 pM and sequenced pairwise as paired-end 100 bp reads. The sequencing platform was an Illumina NovaSeq 6000 with a sequencing depth of >25 million per sample. The read quality in the fastq-files was assessed with ngs-bits (v.2021_03-101), to identify sequencing cycles with low average-quality, adaptor contaminations or to identify repetitive sequences from PCR-amplification. Fastq files were analyzed with Galaxy (https://usegalaxy.eu/) using fastQC and then pairwise aligned with the HPV49 genome by HISAT2 (paired-end). Splice alignment was done using the following parameters: penalty for canonical splice sites, 0; penalty for non-canonical splice sites, 12; penalty function for long introns, *f*(*x*) = −8 + 1*log(*x*); minimum intron length ,20; and maximum intron length, 500.000. Alignments were merged in Galaxy (“merge BAM files “) and then visualized with the Integrative Genome Viewer (version 2.8.0 [29]). The Sashimi blot was created with the Integrative Genome Viewer with a junction minimum of 100 for the merged bam-files and a linear scale at the y-axis. In addition, read coverage and splicing of HPV49 were analyzed and visualized with Geneious Prime (version 2022.0.2).

### Cell culture

NHK were isolated from human foreskin and cultured as previously described (19). The procedure was approved by the ethics committee of the medical faculty of the University Tuebingen (6199/2018BO2) and done according to the principles of the Declaration of Helsinki. Transient transfection using re-circularized HPV genomes and immortalization assays with genomes were carried out as previously described (19). For immortalization assays, retroviral transfer vectors expressing HPV49 E6, E6 co, or E6 SD217 mt and HPV49 E7 were used to infect NHK. Drug selection was carried out with 0.5 µg/mL puromycin and 150 µg/mL G418 for 2–4 days. HPV49 E6/E7 keratinocytes were maintained in E-medium and mitomycin C-treated NIH3T3 J2 cells as described for HPV49 genome-positive keratinocytes (19). C33A cells were cultured in Dulbecco’s modified Eagle’s medium supplemented with 10% fetal calf serum and gentamycin.

### Growth curves

To obtain growth curves of the HPV49 *E6-* and *E7*-expressing cell lines, 5 × 10^4^ cells were seeded into six-well plates, and the number of living cells was determined with an automated cell counter (Invitrogen Corporation, Countess automated cell counter).

### Immunoblot analysis

Transfected cells were harvested 48 h post transfection and lysed in 1% Igepal CA-630, 1% sodium deoxycholate, 0.1% SDS, 150 mM sodium chloride, 10 mM sodium phosphate pH 7.2, 2 mM EDTA, 50 mM sodium fluoride, 1× complete EDTA-free protease inhibitor (Roche), and 1× PhosStop (Roche). E6 proteins were detected by an anti-HA antibody (Cell Signaling, rabbit mAb, 3724) and HSP90 (mouse mAb, Santa Cruz, sc69703) served as a loading control.

### Recombinant plasmids

The pGEM4-HPV49 plasmid and the E8- genome have been previously described (19). HPV49 SD217 mt and HPV49 E8-/SD217 mt genomes harbor exchanges of nt. 217 and 220 (AGGTA to AAGTT), which are silent in *E6*. The complete HPV49 *E6* gene was codon optimized (E6 co) and synthesized by GenScript. The HPV49 E6, E6 co, and E6 SD217 mt genes were cloned into the BamHI restriction site of a pSG5-3xHA vector (based upon pSG5 [Stratagene]). Retroviral transfer plasmids pLXSN-neo HPV49 E6, E6 co, or E6 SD217mt are based upon pLXSN-neo (Clontech), and inserts encoding *E6* were cloned into the BamHI restriction site. PMSCV-puro HPV49 E7 is based upon pMSCV-puro (Clontech), and the HPV49 E7 sequence was inserted between the BglII and EcoRI restriction sites. The pSG5-HPV49 SD217/SA3281 plasmid harbors HPV49 nt. 149–217/3,281–3,502 and was obtained by cloning reverse transcription-PCR fragment after adding restriction sites and used as a copy number control. The inserts of all plasmids were validated by DNA sequencing and in the case of the pSG-HPV49 E6 expression vectors by complete plasmid sequencing to exclude additional mutations (Eurofins Genomics).

### Quantitative PCR

Total RNA was isolated from transfected keratinocytes or HPV-containing cell lines. Where indicated, polyA^+^ RNA was enriched from total RNA as previously described (19). cDNA was synthesized using the QuantiTect RT Kit (Qiagen), and 50 ng cDNA was used per reaction using a LightCycler480 system and the LightCycler480 SYBR green Master mix (Roche). A thermal profile of 10 min at 95°C followed by 45 cycles for 10 s at 95°C, 15 s at 55° or 60°C (depending on the primer pair), and 15 s at 72°C, followed by a melting curve analysis was used. Data were acquired and analyzed using the LightCycler 480 software program, version 1.5 (Roche Applied Science). Primer sequences for *PGK1* (phosphoglycerate kinase 1) and HPV49 *URR^E2*, *URR^E4*, *E1^E2*, *E1^E4*, *E8^E2*, *E6*, and *E7* have been previously described (19). HPV49 E6 co was detected with HPV49 E6 co F (TTTCAACCTGCTGTGGAAGG) and HPV49 E6 co R (TTTGTGAACTCGTGGTAGGC). HPV49 SD217/SA3281 was detected with CGCTTGCGTGCTGTACTTT (forward) and GAGTTGGAGGCTGCTGTAGG (reverse) and HPV49 *E1* with CATGCAAAGAGTAGAGAAACTGTTG (forward) and ACACAGATGAGTCCATACTGCC (reverse) primers. Copy numbers were determined by known plasmid standards analyzed in parallel.

## Data Availability

Data sets have been deposited in the NCBI Sequence Read Archive (SRA) under BioSample accession PRJNA1199202.

## SUPPLEMENTAL MATERIAL

Figure S1 jvi.01640-24-s0001.pdf SD217 cryptic splice acceptors in pLXSN.

ASM does not own the copyrights to Supplemental Material that may be linked to, or accessed through, an article. The authors have granted ASM a non-exclusive, world-wide license to publish the Supplemental Material files. Please contact the corresponding author directly for reuse.
