# Supplementary material for: A splice donor in E6 influences keratinocyte immortalization by beta-HPV49
Source: J Virol. 2025 Jan 22;99(2):e01640-24. doi: 10.1128/jvi.01640-24 (PMC11852731; doi:10.1128/jvi.01640-24)
Supplement: Figure S1 — SD217 cryptic splice acceptors in pLXSN. [file jvi.01640-24-s0001.pdf]

Supplemental Figure 1

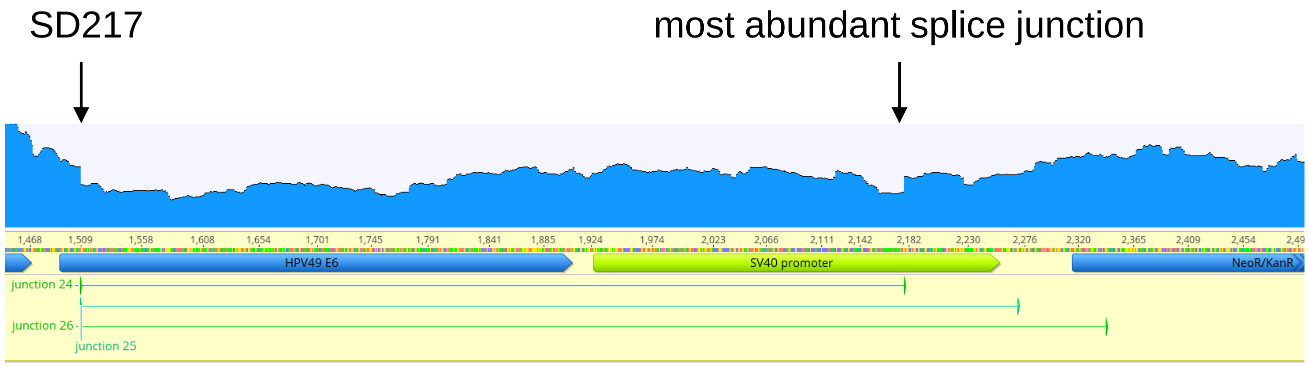

Figure S1:
RNA-seq reads mapped to the pLXSN-HPV49 E6 expression vector derived from a stable HPV49 E6- and E7-expressing keratinocyte cell line. Splice junctions using SD217 and three different cryptic splice sites in the vector backbone were identified. Arrows indicate the position of HPV49 SD217 and the most abundant splice acceptor site (1051 reads) in the vector backbone. Read mapping and graphic representation were generated with Geneious Prime software version 2022.0.2.
